# Marginal notes, June 2025. Artificial Stupidity

**DOI:** 10.1099/jmm.0.002049

**Published:** 2025-07-25

**Authors:** Timothy J. J. Inglis

**Affiliations:** 1 Schools of Medicine and Biomedical Sciences, University of Western Australia, Crawley, WA 6009, Australia; 2 PatWest Laboratory Medicine WA, PP Building, QEII Medical Centre, Nedlands, WA 6009, Australia

**Keywords:** artificial intelligence, clinical microbiology, diagnostic heuristic, diagnostic methods, disease ontology, laboratory algorithm

## Human-scale science

The ever-increasing presence of artificial intelligence (AI) and its near-neighbour species has become a given in every area of infection science. I have to admit some responsibility for this, having been an early adopter of machine learning and an advocate for digital literacy over the last decade. AI, no doubt, poses a set of challenges to scientific journal publishing [1]. The ethics, governance and data linkage regulation of AI are coming into view and have become the subject of robust professional debate, given dwindling trust in biomedical science among Western policy makers. This year’s ESCMID (European Society for Clinical Microbiology and Infectious Diseases) Global conference in Vienna took an important first step in forming a study group on AI and set itself some bold objectives around digital literacy. Since then, a thoughtful paper in the online journal *Aeon* discussed the broader issue of the predicted end of intelligence: the point at which human intelligence surrenders to the work of information machines [2]. This somewhat apocalyptic view of the datascape was predicted decades ago but, in recent years, has gained credibility through the proliferation and adoption of AI applications.

## Keeping up with the pace of progress

While we still have agency in the hands-on work of data implementation, the integration of this technology into real-world clinical microbiology and infectious disease reveals key areas of human effort that add value to our algorithms. For example, I'm impressed how colleagues with a decade or so of specialist training and clinical experience are able to intuitively adapt rigid laboratory workflows to changing demands on a clinical laboratory with only limited data. While large language models may be able to scan the contents of the World Wide Web in seconds and summarize what’s out there in the dataverse, they lack your training and experience in how to frame the question, interpret the answer, set it in a practical context and work out the implications for governance, training and audit. AI doesn't yet seem capable of responsibly handling the outliers that are overrepresented in the day-to-day clinical microbiology workload. This phase of AI integration will not last for long, but for now, there’s value in understanding how best to employ AI as a complementary technology. Hence, we are interested in supervised machine learning [3,4].

## Dynamic worldview

Stepping back from the screen, it’s worthwhile to run a quick inventory check on the major milestones in specialist training, credentials and qualifications that form the foundation of subject-matter expertise. That rational, scientific worldview is dynamic and subject to revision when illuminated by new insight. For those at the senior end of their careers in infection science, the past decades have seen an explosion in molecular bioscience, computational biology, integrated systems biology and the application of these to public health medicine. Integration of fundamental concepts such as biocomplexity into frontline clinical microbiology and infectious disease practice has brought narrative continuity to those who stay up to date with new learnings [5].

## More than an algorithm

This journal’s editorial group and the wider Microbiology Society’s membership are an important part of that community of practice, as we seek to understand structure, function and significance in our corner of the natural world. As previously explored [6], this rational scientific worldview generates a means of classifying micro-organisms and the diseases they cause. The logical consequence of this classification or ontology is a rational process or heuristic for dealing with those diseases. Before we equate heuristic with algorithm, let’s remember that an algorithm you develop, for example, to predict positive blood cultures [7], requires coding, assembly, training, development, statistical evaluation of test performance, refinement and formal validation, plus the required alignment with regulatory standards. The consequences of blindly applying somebody else’s algorithm to your own clinical laboratory output without the above steps in correct sequence underline the difference between heuristic and algorithm. As a heuristic follows on from its corresponding ontology, in the case of blood culture outcome prediction, the heuristic cannot deliver its intended outcome if it has been disconnected from its underlying ontology. In the specific case of blood culture prediction, the algorithm cannot predict the bacteria identified in the blood culture if the non-microbiological attributes of the disease do not differ between causal bacterial species (the emerging theme of disease phenotypes was explored in our recent paper on the subject [8]).

## Lost in translation

On a lighter note, there is a difference between data linkage and integration of AI into medical microbiology. Large language models are one of the technologies coming into general use through web browsers and computer operating systems. On a recent trip overseas, our driver used Google Translate to provide a running commentary on sights we drove past, using Australian English for the output. The result was a reasonable stab at Okker Strine, with occasional throwbacks to Banjo Paterson’s era. However, it was all delivered in an American accent, and every comment ended with an ‘, eh?’ pronounced as ‘E…H’. In our field, the productive co-existence of AI and human intelligence is not a given. Ignorance is not the same as stupidity. The above illustration is an example of ignorance of Aussie kultcha. However, the deliberate denigration of decades of biomedical science, while favouring strident social media influencers, is much worse. As any clinical bacteriologist knows, you have to choose your selective media very carefully.

Tim Inglis, co-EiC.

## Abbreviations

AI: artificial intelligence
ESCMID: European Society for Clinical Microbiology and Infectious Diseases
